# A Mixed-Methods Feasibility Study of Integrated Pediatric Complex Care: Experiences of Parents With Care and the Value of Parent Engagement in Research

**DOI:** 10.3389/fresc.2021.710335

**Published:** 2021-10-28

**Authors:** Oksana Hlyva, Charlene Rae, Shelby Deibert, Rakhshan Kamran, Haniah Shaikh, Lehana Thabane, Peter Rosenbaum, Anne Klassen, Audrey Lim

**Affiliations:** ^1^Department of Pediatrics, McMaster University, Hamilton, ON, Canada; ^2^Michael G. DeGroote School of Medicine, McMaster University, Hamilton, ON, Canada; ^3^Department of Health, Research Methods, Evidence & Impact, McMaster University, Hamilton, ON, Canada

**Keywords:** qualitative, mixed methods, feasibility study, children medical complexity, complex care, patient engagement

## Abstract

**Introduction:** Children with medical complexity (CMC) are among the most vulnerable children in society. These children and their families face challenges of fragmented care and are at risk for poorer health outcomes. Families with CMC play a vital role in providing care and navigating the complexities of healthcare systems. It is essential to understand the best ways to engage these families in research to improve the care and optimize the health of CMC.

**Objectives:** This study explored parent engagement within the context of a feasibility study evaluating an Integrated Tertiary Complex Care (ITCC) clinic created to support CMC closer to home. This paper aimed: (1) to understand the family experiences of care and (2) to explore parent engagement in the study.

**Method:** This mixed-methods feasibility study included three components. First, feedback from focus groups was used to identify the common themes that informed interviews with parents. Second, one-on-one interviews were conducted with parents to explore their experience with care, such as the ITCC clinic, using an interpretative description approach. Third, the questionnaires were completed by parents at baseline and 6-months post-baseline. These questionnaires included demographic and cost information and three validated scales designed to measure the caregiver strain, family-centered care, and parental health. The recruitment rate, percentage completion of the questionnaires, and open-ended comments were used to assess parent engagement in the study.

**Results:** The focus groups involved 24 parents, of which 19 (14 women, five men) provided comments. The findings identified the importance of Complex Care Team (CC Team) accessibility, local access, and family-centered approach to care. The challenges noted were access to homecare nursing, fatigue, and lack of respite affecting caregiver well-being. In this study, 17 parents participated in one-on-one interviews. The identified themes relevant to care experience were proximity, continuity, and coordination of care. The parents who received care through the ITCC clinic appreciated receiving care closer to home. The baseline questionnaires were completed by 44 of 77 (57%) eligible parents. Only 24 (31%) completed the 6-month questionnaire. The challenges with study recruitment and follow-up were identified.

**Conclusion:** Family engagement was a challenging yet necessary endeavor to understand how to tailor the healthcare to meet the complex needs of families caring for CMC.

## Introduction

Children with medical complexity (CMC) are among the most vulnerable children in society. They have multiple chronic conditions and significant functional limitations and are dependent on life-sustaining technology, such as tracheostomy, home mechanical ventilation, and enteral feeding tubes for daily survival (1–3). CMC are at very high risk of multiple and prolonged hospitalizations, frequent medical errors, and poor health outcomes (4, 5). CMC comprise only 0.67% of all children in Ontario (6), but this group utilizes about one-third of all the provincial child health resources, thereby having a substantial impact on the healthcare system (1, 7, 8). A study from the Canadian Institute for Health Information reported that over a 2-year period, the hospital costs associated with children and youth with medical complexity were $866 million (9). CMC accounted for 37% of all hospital admissions and 54% of total days in the hospital (9). Similarly, the impact of caring and coordinating complex, fragmented care for such children is substantial for the families.

Care for CMC requires close monitoring by multiple healthcare providers (10–12). Different care delivery models exist for CMC, such as primary care-centered frameworks, consultative or co-management-centered models, and episode-based models (10). The primary care-centered frameworks can be based in the community or tertiary care centers and focus on providing coordinated care for CMC through a dedicated primary care center (10). These models, where offered, serve as the first entry-point for CMC to access the healthcare (10). The co-management-centered models, on the contrary, are not the first entry-point for CMC and encompass subspecialty providers in tertiary centers coordinating care with primary care providers in the community (10). Last, episode-based models focus on providing care during a discrete and acute period of illness and are usually time-limited in nature (10). Examples of episode-based models include transitional care homes where CMC are between hospitalization and home. The successful complex care programs at tertiary care centers deliver better care at a lower cost due to a reduction in preventable inpatient and emergency department visits (12–15).

Despite the benefits of different models of care, CMC and their families still face barriers when accessing the complex care programs in tertiary centers. Since many children do not live near the tertiary care centers (16), traveling to these centers can present financial, physical, and social challenges for the caregivers and their CMC. Further, poor communication across the healthcare settings may limit the appreciation of tertiary healthcare providers of the breadth of community services that can provide additional support to CMC (10, 17). The strategies to overcome these barriers include creating enhanced primary care center-based complex care programs dedicated to care for CMC with resources and staff centralized at a tertiary center, care coordinators, and standardized care coordination quality improvement tools (1, 10, 14, 15, 18, 19). Integration of a tertiary care center with a community-oriented pediatrics team, different from the previous CMC care models, has been shown to provide cost-saving benefits, increased family-centeredness, decreased hospitalization rates, decreased parental work loss, and higher family and healthcare provider satisfaction (20–22).

The Integrated Tertiary Complex Care (ITCC) clinic is the first clinic of its kind where a tertiary complex care clinic is embedded in a treatment center of children. The ITCC clinic is located within the Niagara Children's Center, 80 km from the McMaster Children's Hospital (MCH), a large tertiary hospital for children (23). Established in 2015, the ITCC clinic is a collaboration between a tertiary academic hospital and regional children's treatment center, created to coordinate, support, and bring care closer to home for CMC. Implemented as a monthly full-day clinic, the MCH CC Team consisting of a pediatrician, nurse practitioner, and respiratory therapist travel to Niagara to conduct clinics in partnership with the allied health and community team at Niagara Children's Center (24). The aim of the ITCC clinic is to provide comprehensive, holistic care for CMC, improving communication between the tertiary, community healthcare partners, and parents of CMC while alleviating the travel burden to a tertiary center. It is important to evaluate the ITCC in comparison with the existing models of care to ensure CMC are receiving optimal care.

To evaluate the ITCC clinic, parent engagement is necessary to capture the perspective of the user of this system. Parent engagement can include consulting, providing information to inform decisions, sharing leadership, and defining agendas (25). It is notable that the higher levels of engagement, such as shared leadership, are not always desired by the patients and families, and not always the most effective, depending on the research and clinical context (25). Increasingly, the researchers and clinicians view parent engagement as an essential way to inform better care and include patient experiences to help balance an unstated bias toward the clinical and system outcomes (25). A recent literature examining the quality of care for CMC has emphasized the need for parent research involvement through consultation to evaluate the implemented improvements in care provision and provide crucial feedback to the providers to facilitate the sustainable changes (26).

A pilot study was conducted to evaluate the ITCC clinic model of care for CMC, reported in a forthcoming paper. This paper outlines the findings of a secondary data analysis of the pilot study, aiming: (1) to understand family experiences of care and (2) to explore parent engagement.

## Methods

This mixed-methods feasibility study involved a Family Engagement Day with focus groups, one-on-one interviews with parents, and the completion of questionnaires. The study took place from October 2016 to March 2018 at MCH and the newly created ITCC clinic within Niagara Children's Center in the Niagara Peninsula. Ethics approval for all aspects of this study was obtained from the Hamilton Integrated Research Ethics Board prior to the recruitment (#1011).

### Participants

The parents of CMC, from two different models of care, within the catchment area of MCH who were followed by the CC Team were recruited for the study. The parents who met the study eligibility criteria and consented to be contacted by the research team were invited to participate. They were subsequently screened for eligibility with the following criteria: they were the primary caregiver(s) to the patient and they could read and understand English. The parents were excluded from the pilot study for reasons that included caregiver hardship (such as the parent being ill or child being acutely ill). An informed written consent was obtained from all the participants through clinic visits or by mail.

### Data Collection

This study is a secondary analysis using data collected as part of a pilot feasibility study evaluating an ITCC model of care. It involved three sources: focus groups, one-on-one interviews, and questionnaires. The data collection methods used in the feasibility study are described below.

#### Family Engagement Day

As part of a Family Engagement Day held in March 2016 at MCH, focus group discussions were facilitated by the CC Team. The focus groups were designed to engage parents and healthcare providers to identify the challenges and facilitators with the goal of improving the delivery of care to CMC. The day involved 24 parents, 24 healthcare providers, and 12 stakeholders (e.g., managers of Local Health Integration Unit (LHIN), CEOs, and school board representatives). There were five breakout groups, each consisting of a facilitator (member of the CC team), parents (ITCC and tertiary care), a healthcare provider, and a stakeholder. The breakout groups were used during the two sessions. In the first session, “Understanding Your Needs – Value Stream Mapping Exercise,” the participants were asked the following: (1) Describe your ideal care and current challenges; (2) What does a typical care journey look like?; and (3) Describe how you feel during your care journey. During the second session, “What Matters Most? Interactive Discussion,” the participants were asked to discuss the key elements for successful complex care and coordination, such as: Medical Care Coordination, Home and Community Care Coordination, Knowledge Building for Families, and Community/Partner Engagement. The key findings in the feasibility study included the needs of families: (1) better communication between inpatient and outpatient services, between agencies, and between the hospital and community services, (2) coordination of multiple appointments, and (3) trained professionals that are competent and willing to care for a child with medical complexity. These findings were used to structure the questions in the interview guide for the next stage of the study. Focus group sessions were audiotaped and transcribed. The manual identification of themes was done by two coders using a line-by-line approach as a unit of analysis.

#### One-On-One Interviews

Recruitment for one-on-one interviews took place from August to December 2017. A purposeful sample of parents was recruited from tertiary hospital catchment regions with a focused representation of parents from the Niagara region. A written consent was obtained at the time of the interview or through mail. Informed by the focus group findings, the aim of the interviews was to gain an in-depth understanding of the family experiences of care at both the tertiary care site and the ITCC clinic, as well as care coordination through the new model of care at the ITCC clinic.

The interviews were conducted by an experienced qualitative researcher who used a semi-structured interview guide. After the initial eight interviews were completed, an interim analysis was conducted to further refine the interview guide. The interviews were transcribed, de-identified, and analyzed using NVivo 10 software. The interviews were coded line-by-line to identify the themes and subthemes relating to family experiences of care and parent engagement. The interviews were conducted until no new concepts were identified. Prior to analysis, the codes and full interview transcripts were reviewed by OH to ensure that coding accurately captured information in the full transcripts. Informed by the interpretive description method, the interviews were analyzed using a rigorous constant comparative and iterative approach to identify and describe the themes and subthemes (27). The results were then shared with the team, discussed, and refined.

#### Questionnaires

During the feasibility study, the parents were invited by phone or in person to complete the questionnaire online or using a paper booklet. Timing of completion was either during a clinic on the day of scheduled appointment of their child with the CC Team, or outside the clinic at the time convenient for the family. All data were entered into a Research Electronic Data Capture (REDCap, Vanderbilt University, TN, USA) database supported by the Department of Pediatrics at McMaster University, ON, Canada. A link to complete the 6-month follow-up questionnaire directly into REDCap was sent *via* email to the parents who provided an email. Those who did not provide an email were given a paper copy of the questionnaire in the clinic.

The questionnaire included demographic questions, collected through standardized survey questions, such as caregiver age, gender, race, marital status, relationship to child, and education. The parents were also asked about their health and medical condition of their child. The impact of the chronic illness of the child on their family was measured using the Impact on Family Scale, a 15-item scale that notably examines the financial burdens as well as emotional concerns for families (28). Parental perceptions of whether the care provided displayed family-centeredness were assessed using the Measure of Process of Care (MPOC-20). The MPOC-20 is comprised of five subscales: respectful and supportive care, enabling and partnership, providing general information, providing specific information, and coordination and comprehensive care (29). The EQ5D-5L (EuroQol Office, The Netherlands) was used to assess the health status of the caregivers. This scale asks about five dimensions: mobility, self-care, usual activities, pain/discomfort, and anxiety/depression (30). A series of questions focused on out-of-pocket costs for medical care were included to assess how well this data could be obtained from the parents. Finally, at the end of the questionnaire, an open space was available for parents to provide any additional comments they had for the research team. The open-ended comments were coded to identify the themes and subthemes relating to family experiences of care and parent engagement.

Parent engagement was further examined in questionnaire data by assessing both the response rates to the questionnaires as well as completion rates of each section of the questionnaire.

## Results

### Aim #1: Family Experience With Care

This section draws on the focus groups, one-on-one interviews, and open-ended comments from the questionnaires.

#### Focus Group and One-On-One Interviews

The focus groups involved 24 parents, 19 (14 women, five men) of which provided comments during the session. The analysis of the focus group sessions identified the importance of available tertiary care providers, a coordinated care plan, local access to care, and a family-centered approach to care delivery. The findings included the challenges related to accessing trained community nursing, social isolation, and lack of respite, all of which contributed to the burden of care and adversely affected the well-being of caregivers. The participants welcomed and valued the integration of complex care services into the community.

One-on-one interviews were conducted with 17 parents (as shown in Table 1). The key themes from the interviews illustrate family experiences related to the following: proximity and continuity of care closer to home, care coordination, accessibility and communication, and family-centered care. Themes 1 and 2 are specific to the ITCC clinic while the continuity and coordination of care were also reported to be valuable to the partcipants receiving their care at the MCH.

**Table 1:** Demographic characteristics of participants in one-on-one interviews (*N* = 17).

**Characteristic**	***N***
**Gender**	
Female	13
Male	4
**Age**	
20–29	2
30–39	7
40–49	6
Missing	2
**Race**	
White	15
Other	2
**Marital status**	
Common law/Married	13
Single/Separated	4
**Relationship to child**	
Biological Mother	12
Biological Father	4
Foster Parent	1
**Education**	
University or College Degree/Diploma	12
Some Post-Secondary (University or College)	3
Missing data	2
**Employment status**	
Employed full-time	9
Employed part-time	4
Receiving social assistance	2
Receiving unemployment insurance	1
Student	1

##### Theme 1: Complex Care—Proximity and Continuity of Care Closer to Home

This theme builds on the findings from the Family Engagement Day focus groups that highlighted the challenges associated with disjointed care, travel, and long-distance appointments. In the one-on-one interviews, the parents whose child received care through the ITCC clinic appreciated the proximity of care to their home and the easier access to the allied health professionals. Receiving care closer to home was reported to have many advantages. The parents appreciated that, in addition to being close, the team came well prepared with the medical supplies and had access to the medical records of their child:

*I think what works for me is that it's close and that it's Dr. [name]. And they …come equipped with quite a few things. So, last time … normal saline… nebules [were] on back order, and … they had some. And they had silver nitrate … that they were able to give us [for granulation tissue]. So, they come well prepared. … And it's nice that they have access to [child's] lab values…so we were able to bring that up … I just love that it's close. It's still 40 min [drive], but it's not an hour and a half, so it's less than a couple hours in the car*. (P1)

Since the proximity of the clinic meant shorter travel times to the appointments, the parents reported easier logistics, requiring fewer arrangements regarding the time taken off work, childcare for other children, packing all the necessary medical equipment, and navigating unfamiliar hospital and parking environments. Having appointments in the community was less disruptive to the lives and schedules of families:

*So, for [child] to go to [the tertiary center], [they miss] a whole day of school… and [they're] already behind, so missing a lot of days, it impacts [them] a lot. … If [child] just has to go up the road… then [they] only miss half a day, if that*. (P16)

Receiving care through the Children's center also meant a unique benefit for children attending the preschool on site. The parents appreciated the convenient access to the allied health professionals at the center, with some parents wishing for an extension of these services for school-aged children as well.

In addition to proximity, the parents emphasized that familiarity of the team with their child made the process of receiving care less overwhelming for the family:

*I love that it's Dr [name]. [With another physician] … I'm having to retell [child's] story, and they don't know [child] … If it's any other physician, then I will ask … to schedule me in when Dr [name] is going to be in Niagara. … I love that it is a team … and … the same nurse practitioner every time*. (P17)

*I know that group of people well…. [the nurse and respiratory therapist]… know the kids well … [and] … all the pertinent questions to ask. It's an easy… process. Easy to remember everything you need to talk about and you get things done*. (P1)

With proximity and continuity came important relationships that allowed for more proactive and timely care, sometimes eliminating the need to go to the Emergency Department (ED) or Emergency Room (ER):

*And just being able to say, “This is what's going on. What do you think?” And for [the physician] to say, “You need to go to the ER or we're running a clinic today. Why don't you come in to the clinic? And we've been able to treat [the sick child] that way. So… having the close relationship and the continuity… is pretty important*. (P1)

Some parents suggested increasing the number of clinic days at the Niagara Children's Center and emphasized the need for access to the trained staff on a regular basis to meet extensive and often unexpected medical needs of their child:

*There's a couple of times [when the child had an episode] … and it's just we've been fortunate enough that it's been a Complex Care day … and the team has been down here. So, we were able just to drive the 7 min to it…* (P14)

Given the complexities of their children, parents often emphasized the vital importance of continuity in their care:

*[Child]'s very complex, and unless you see [them] often… And I know… we are one of very many patients part of the Complex Care team. So, it's really hard to invest in your patients when you see them a few times a year. With that said, I think that we get excellent care*. (P17)

The continuity of care, consistency, and expertise were valued more highly than proximity of the clinic to their home, so that some families would still be prepared to take the drive to the tertiary center to be seen by the CC Team familiar with the medical complexity of their child.

##### Theme 2: Care Coordination

Having experienced fragmented care, the parents overwhelmingly expressed their appreciation for care coordination through the CC Team. A complex care system navigator scheduling and coordinating appointments with various subspecialty clinics made a significant difference: It minimized travel to the hospital and distress to children and their family.

Given the systemic challenges parents often face with care coordination, the parents attending the ITCC clinic appreciated the simplified process and the effort of team to coordinate and streamline the complex care of the child despite the inherent challenges:

*They try to make things easier when they can. Dr. [name] is always pushing to try and get appointments together. [Doctor] would like to see me not have to travel as much*. (P1)

Care coordination by the CC Team reduced the stress in very important ways. For example, having all involved healthcare providers communicate and synchronize their plans at the ITCC clinic provided a comprehensive holistic approach to care and was reported to be very helpful to families:

*They can… share information with therapists at the Children's Center as well. …Any issue, they can speak with each other about it …to come up with a plan. And it's not me having to go and try and find things out… get information from this person here, and then get information from this person there. They're all linked together. So, everybody is in sync. It's less stressful*. (P16)

Care coordination was often described as easier to manage, less time intensive, and less anxiety-provoking.

##### Theme 3: Accessibility and Communication

All parents agreed that different communication options, such as phone, text, or email made the CC Team members more accessible. In the group followed at the tertiary hospital, parents described access to the CC Team as excellent and the team as very responsive and accommodating, often allowing for parents to receive the clinic appointments when needed and allowing for same-day scheduling. Access to the team reduced the ED visits and other urgent care appointments for parents in both the tertiary hospital and the ITCC groups. Being able to reach the team over the phone often allowed parents to get faster care, or to determine if travel to the hospital was indeed necessary. Some parents valued the opportunity to send pictures and get professional guidance with advice that allowed them to manage the situation on their own. Access to the team minimized the need to travel, and reduced the stress and healthcare resource use for parents:

*If I run out of a prescription or something wasn't [available]… Sometimes… I'll call to reorder a med, and they just don't send it. … The names on the bottle is often different doctors, right, and then it doesn't get to that same doctor. So, I know that I can always text and … it gets taken care of within 24 h. … So, that's extremely helpful. That takes a lot of stress and pressure off… otherwise it would be visit to come in and get a new prescription, ‘cause you … can’t go without it*. (P1)

Understandably, since many parents described travel with their child as stressful, costly, and emotionally exhausting, the parents valued the ability to access care virtually whenever it was possible.

##### Theme 4: Family-Centered Care

All parents agreed that the CC Team provided family-centered care and appreciated the relationships they had with the CC Team. For many parents, being validated and treated by the team as partners in the care and decision-making of their child was very important:

*It's more… personalized. And you feel…included and … valued and you feel like, “I'm not just like a nobody here. I matter to them too. Or my opinion matters here too.” … They do a really good [job] in making sure that you feel like part of the team*. (P16)

Personalized care also meant the CC Team attended to the social and educational needs of the child, and not only their medical needs. For example, the CC Team offering to do a school visit was not expected, but highly appreciated by one parent:

*Right now I'm emailing back and forth with [doctor] to see if we can organize a school visit where … the team can come into [child's] class and answer… any questions students might have, and help in the social aspect … in school. So, even little things like that. … I would have never thought that that's a service that they provide… you only think it's just… medical. But they go far more than just the medical*. (P16)

The parents who attended the ITCC clinic described the team as responsive, respectful, and understanding, giving them time to ask questions and acknowledging that their child may present differently. Such understanding often translated into flexible thinking and treating each child as an individual first:

*I never feel rushed. I always feel like I can ask as many questions as I want. I never feel … that they're watching the clock and they're waiting for the next patient to come in, which I really do appreciate. And I always feel that we are asked how things are going and what we think is going on, because [child] presents very differently. For an ear infection, [child] is not pulling at [child's] ear or rubbing it … [child] usually doesn't spike a temp. [child] is usually throwing up because it's causing [child's] gag reflex to be all out of whack. So, they respect our opinion*. (P17)

The positive relationships with physicians, nurses, and staff were frequently reported to be a very important aspect of family-centered care throughout all the interviews. The parents highly regarded the responsiveness and accessibility of the CC Team to the needs of their child:

*The relationship with the doctors here is excellent. I can always rely on them… to get the kids what they need. … I can always call when I have a problem*. (P1)

The parents felt grateful and often emotional when describing the team members as “saint[s]” and “guardian angels” who are “passionate about the patients” and show that they value their child by “treating their child as gold”:

*Doctors and people involved is that … five percent of people within their field who are not only experts, but they are empathetic. They get it. They understand your life is not typical … I … feel really fortunate that a lot of the five percent people have come into my life. … I always say that if I could change any… everything for [child], I would in a heartbeat, but I would… [crying] I think I would feel lost because I've met so many great people because of [child]*. (P17)

#### Open-Ended Responses From the Questionnaire

In total, 21 parents chose to provide open-ended comments *via* free form text at the end of the questionnaire.

In terms of family experience of care, most comments expressed appreciation of the parents for the care their children received through the CC Team. The team was described as “professional,” “supportive,” “caring,” “kind,” and “exceptional.” The parents reported that they trusted the knowledge and expertise of the team about the care of their child and felt listened to and not rushed during their appointment. The parents were grateful for the help of the team with their concerns, coordination of appointments in an accommodating way, and how the team celebrated the milestones of their child' with enthusiasm. The parents appreciated how the team tried to make their lives easier and were understanding of their circumstances, which they noted was not the case outside of the complex care clinics. Overall, the parents highlighted that it was difficult advocating for their child on their own and they underscored the role of the CC Team beyond a “transactional” one. Some parents were grateful to the team for teaching them how to care for their child during very difficult times, whereas others attributed reduced hospital visits and the success of their child directly to the expert guidance of their CC Team. An example quote from a follow-up questionnaire shows the support from the perspective of a parent:

*The complex care team is by far the most effective and competent team that we've dealt with over the past couple of years. You can tell they care and are invested in helping us make our child's health as best [as] it can be. They also work collectively to ensure there aren't gaps in our child's care. They go above and beyond to make sure we have what we need to care for our child and they give us hope that this is what the health care system could look like if everyone invested the same time and energy into their patients*. (P46)

### Aim #2: Parent Engagement in Research

#### Focus Group and One-On-One Interviews

In total, 24 parents of CMC were invited to attend the focus group, with 19 actively participating contributing comments in the transcripts. For the one-on-one interviews, 17 parents participated, with interviews lasting between 60 and 90 min.

In the one-on-one interviews, the parents viewed their engagement in research as validating their complex lives and as an indicator of family-centered care.

*Doing these type of studies … of trying to figure out how it's affecting us. … It just goes to show how family-centered it really is*. (P16)

#### Questionnaires

In total, 49 (64%) of 77 eligible primary caregivers consented to complete a questionnaire. Of these, 44 (57%) completed the initial questionnaire and 24 (31%) completed the 6-month follow-up questionnaire. The initial sample included four parents of children who attended the ITCC clinic, of whom two also completed the follow-up survey. The demographic characteristics for the sample are shown in Table 2.

**Table 2:** The demographic characteristics of participants in the questionnaire study.

**Characteristic**		**Assessment 1**		**Assessment 2**	
Age (mean (SD))		37.3 (6.8)		36.7 (7.2)	
		***N***	**%**	***N***	**%**
Region	Hamilton or other	40	90.9	22	92
	Niagara	4	9.1	2	8
Gender	Female	39	88.6	19	79
	Male	5	11.4	5	21
Race	White	39	88.6	20	83
	Other	5	11.4	4	17
Relation to child	Biological Mother	35	79.5	19	79
	Biological Father	5	11.4	5	21
	Foster Parent	4	9.1	0	0

For the three survey tools that were administered, the sub-scale and overall scores were obtained for the majority of parents (≥92%), with the exception of two sub-scales of the MPOC. The MPOC sub-scales of “Providing General Information” and “Providing Specific Information,” during the initial assessment, had slightly lower completion rates of 82 and 86%, respectively. Table 3 provides summary statistics by assessment for the questionnaires.

**Table 3:** Summary statistics for the Measure of Process of Care (MPOC), EQ5D, and Impact on Family measures.

	**Initial Assessment (*N* = 44)**			**Follow-up Assessment (*N* = 24)**		
	**Mean**	**SD**	**% complete**	**Mean**	**SD**	**% complete**
**MPOC**						
Respectful & Supportive Care	5.7	1.1	95	6.1	1.0	96
Enabling & Partnership	5.6	1.1	95	6.0	1.1	96
Providing General Information	4.0	1.7	82	4.0	1.6	96
Providing Specific Information	4.6	1.8	86	5.0	1.7	96
Coordination & Comprehensive Care	5.5	1.2	95	5.9	1.2	96
**EQ5D**						
EQ5D Overall Score using CDN valuation	0.8	0.11	95	0.8	0.1	92
**Impact on Family**						
Impact on Family Burden Score	43.8	9.1	95	45.1	8.2	96
Impact on Family Financial Burden Score	14.4	4.0	95	14.4	3.0	96

To capture the cost-related data proved challenging. The completion of cost data ranged from 78 to 96% for parents reporting out-of-pocket costs. The costs were reported for visits to the emergency room, community pediatrician, and family doctor, as well as hospital admissions. The costs provided included transportation [bus, taxi, and personal vehicle (km)] and parking. The lowest completion rate at both assessment points was cost associated with visits to the community pediatricians. Prescription medication used by the child was reported by 34 and 18 parents at the initial assessment and follow-up, respectively. The complete cost data for both prescription and over-the-counter medication was reported as high (>90%) at both the initial and follow-up assessments. For devices and supplies at the initial assessment, 36 parents reported purchasing devices and supplies related to care, with nine having full coverage of costs by insurance. Of the 27 parents reporting out-of-pocket costs, 21 provided costs estimates. At the follow-up assessment, the completion rates for device and supply costs increased with 20 parents reporting this cost, and five reporting full insurance coverage. In total, 14 of 15 parents with out-of-pocket costs provided cost estimates with this variable. The overall costs could be calculated for all the questionnaires. However, only 32 of 44 and 18 of 24 parents at the initial and follow-up assessments, respectively, had complete cost data for all the elements. The remaining parents had at least one area of missing cost data, therefore the total cost value calculated might not be reflective of their experience.

Furthermore, the detailed open-ended comments from 21 parents provided insight into various areas of care as well as the questionnaire administration.

Challenges with the questionnaire completion identified by the parents related to time constraints and applicability of the questions to their family. The parents dedicated time to complete the questionnaire because they felt passionate about helping with the study and improving the care for the patient population. Some respondents indicated they would have preferred to engage in a conversation over the phone or in person, instead of completing a questionnaire. Feedback in the baseline questionnaires suggested that certain questions pertaining to costs needed to be “time specific” and to consider existing funding models:

*If I was asked to complete this survey every year of [child's] life from birth to now, they would all look very different (far more medical appointments in [child's] first year–over 120)*. (P41)

*This survey would be easier and more accurate if you did a yearly overall expense of medical supplies and found out how much ADP [public] funding parents received…as opposed to individual items like catheters etc*. (P3)

Finally, the parents pointed out the unique circumstances of their family that made some questions not applicable (e.g., foster parents, parents who work full-time, and parents who work flexible hours). Some alluded to multiple complex factors in addition to the illness of their child's which “made some questions difficult to answer” and reminded the research team that “extraordinary expenses and circumstances do not fit in boxes but happen nonetheless.” Some parents emphasized the often overlooked “opportunity cost” when the promised services and funding failed to deliver.

## Discussion

This mixed-methods pilot study focused on the first integrated complex care clinic embedded in a children's treatment center that was implemented to provide multi-disciplinary complex care closer to home for CMC and their families. The ITCC clinic provided coordinated care for CMC and alleviated the burden of travel for families. Access to specialists trained to understand the comprehensive needs of their children in the community was an important aspect of the model of care to meet the continuous, intense needs of their children. Building on the knowledge gained from the Family Engagement Day focus groups, interviews, and questionnaires was used to understand family experiences of care and explore the parent engagement in the research study. Further, the data collected provided important information on the best ways to engage these families in research to improve care and optimize health of CMC. The parental perspectives provided valuable insights into the experience of their families with care, further demonstrating the importance of parent engagement in research (25, 26). The finding in our study regarding the parent capacity to fulfill the multitude of roles and tasks that go with caring and care coordinating for CMC is consistent with the other studies (31, 32). A recent systematic review suggests a growing body of research focusing on the health and well-being of CMC parents as primary caregivers (33). A scoping review on the interventions to improve the health and well-being of parents of children with special healthcare needs calls for careful tailoring to ensure that such interventions are both feasible for delivery within routine care settings, as well as relevant and accessible (34). The present study contributes to our understanding of feasibility in conjunction with the relevance and accessibility to families of CMC; however, future studies are needed to understand the feasibility and tailoring of interventions aimed to further alleviate the caregiver burden.

The care experience of the parents with the CC Team was overwhelmingly positive. The parents who were within the ITCC model of care reported to have reduced stress, disruption of daily life, and less travel time. The parents were very appreciative of the decreased burden related to care coordination and advocacy for multifaceted needs of their child, such as social and educational needs. Continuity, accessibility, and positive personalized relationships with the CC Team members were very highly regarded and valued. The parents felt heard, valued, and supported by the CC Team as partners in care of their child.

The information and communication were found to be important aspects of family experience. Improvements in the communication between the CC Team and allied health professionals were reported to have enhanced the management of CMC. Additionally, the parents indicated a strong need for proactive communication, with collaboration among all the stakeholders across different systems. This finding is consistent with other research on the vital importance of communication for parents of CMC (35) and other populations where the institutional policies regarding privacy adversely impacted the communication flow among all the stakeholders. In this respect, our pre-pandemic study illustrating the importance of accessibility *via* different communication modes is relevant to the present world of virtual care and is aligned with the call for a new “normal” in the post-pandemic care delivery (36). This new normal would include expanding the range, nature, and locations of services and supports for families as well as hybrid blended care delivery models since families still value hands-on, relationship-based, and personalized approaches.

The cost-related impact of caring for CMC is a relevant area of family experience. In the open-ended responses, the parents emphasized that extraordinary costs and lost opportunity costs cannot be captured adequately through the quantitative data. Our study underscores the extraordinary costs, which are not always medical and are associated with parenting and ensuring quality of life and optimal health outcomes for CMC. A self-directed funding and understanding what goes into the cost of raising a child with medical complexity would be important steps toward a positive change.

In addition to the intense involvement of parents in the care of their child, parents shared insights pertinent to our understanding of their engagement in research. The parents appeared to be more engaged in qualitative compared with the quantitative data collection, as demonstrated by the response rates and completion of interviews. The Family Engagement Day was very well attended and was an effective method of engaging parents in shaping the next phases of the feasibility study that include the design of interview guides. While the questionnaires had a high rate of completion, overall quantitative data collection was challenging. Recruitment rates and consent rates for the study were low, and it was difficult to engage parents to complete the follow-up questionnaires. It was evident from the qualitative data that loss to (research) follow-up was likely due to time constraints, caregiver fatigue, or limited applicability of the questionnaire items to their child. The open-ended responses of the questionnaire offered useful insights and revealed the amenability of parents to qualitative over the quantitative data collection. Furthermore, the length of the interviews (up to 90 min) points to a possible preference for more personal, narrative, and reflective forms of engagement. The mixed-methods approach was useful to explore the areas of care deemed important by parents but not captured within the questionnaire design. It also allowed for some insights into the parental engagement within different research methods. It would be useful to further integrate the design of the questionnaire used in the quantitative data collection into Family Engagement Days and one-on-one interviews. This would help to ensure that the questionnaire developed is capturing data in a way that is meaningful for parents, potentially improving the response rates. Their involvement in the questionnaire design may help to improve the response rates by making the questions more meaningful to them, and easier for them to complete.

In addition to building research capacity to capture the complex “story books” of the families (37), the balance with regard to the extent and sustainability of parent engagement should be explored in the future studies. It is known that the families of CMC are consumed with managing health of their child, which often limits their ability to engage in research (38). The multitude of often-invisible roles and tasks parents perform as caregivers, and considerations for complex daily realities of these families require further exploration. While patient and family engagement has been around for the last 20 years, the relationships of the researchers with families are still in their early stages (22). More studies are needed on the impact of patient engagement on research (39) and care, as well as guidance on engaging patients and their families (22, 40).

Several limitations of this study merit consideration. First, recruitment for the questionnaire survey was low for several reasons. Some parents were ineligible for the study due to language, social considerations, and burden of illness/stress in this population. Of eligible parents, some parents were not available for the study recruitment discussions in the clinic prior to or following the appointment of their child and others approached in the clinic did not want to take part due to already prolonged duration of complex care appointments. The transition to telephone recruitment proved effective and moderately improved the recruitment rates. Although we invited all the eligible parents on patient lists to participate in this study, the perspectives of some parents might have been missed as it is likely that those parents that are most engaged participated. Future research should aim to explore diverse methods of recruiting and engaging with parents who might not regularly participate in the research. Second, very few parents were recruited from the ITCC clinic (5 of 17 parents). A larger sample is needed from this clinic to capture a wider perspective from the parents to prevent potential bias. Third, even though the questionnaires were generally well-accepted, a few parents felt that some questions did not apply to the unique circumstances of their family, thus suggesting qualitative means may allow more nuanced ways to capture the temporal, contextual, and individual variability.

## Conclusion

Overall, the family experience of care was generally positive for the parents of CMC. In particular, the ITCC clinic model of care offers a positive experience for the CMC and families. The ITCC clinic provides CMC and families with holistic, multidisciplinary healthcare close to their home communities, which minimizes disruptions due to travel burden and offers coordinated care between the specialists from a tertiary center and community care providers. Even though the sample size was small, it appears that the models of care may have substantial influence on the experience of care of parents. Parent engagement in research through qualitative methods allowed for richer data collection and the ability to capture information, which might have been missed through a survey. Open-ended response options in the surveys provide a means of improving survey-based engagement methods. However, we found that the participant engagement remained low despite including open-ended response options in our survey compared with the qualitative components of our study. Knowing how best to engage families of CMC in research studies is necessary for future research to understand how to evaluate and tailor the healthcare to the complex needs of families caring for CMC.

## Data Availability Statement

The original contributions presented in the study are included in the article/supplementary material, further inquiries can be directed to the corresponding author/s.

## Ethics Statement

The studies involving human participants were reviewed and approved by Hamilton Integrated Research Ethics Board. The patients/participants provided their written informed consent to participate in this study.

## Author Contributions

OH analyzed the qualitative data, drafted and edited the manuscript based on the critical feedback. HS, RK, CR, and SD assisted with the data analysis and writing of the paper. PR involved at various stages in discussions about the design of the study and contributed to writing the final paper. AK assisted in the design of the study, the analysis, and critically reviewed the paper. LT provided statistical leadership, contributed to the design, and supervision of this study. AL contributed to the conception and design of the study, interpretation of the data, and revising the manuscript and edited the final version for submission. All authors contributed to the article and approved the submitted version.

## Funding

This study was funded by a grant from the Hamilton Health Sciences Foundation.

## Conflict of Interest

The authors declare that the research was conducted in the absence of any commercial or financial relationships that could be construed as a potential conflict of interest.

## Publisher's Note

All claims expressed in this article are solely those of the authors and do not necessarily represent those of their affiliated organizations, or those of the publisher, the editors and the reviewers. Any product that may be evaluated in this article, or claim that may be made by its manufacturer, is not guaranteed or endorsed by the publisher.
